# Ayurveda Herbal Medicine-induced Liver Cirrhosis

**DOI:** 10.7759/cureus.4122

**Published:** 2019-02-22

**Authors:** Cyriac A Philips, Philip Augustine, Rajaguru Paramaguru, Rizwan Ahamed, Guruprasad Padsalgi

**Affiliations:** 1 Gastroenterology, Cochin Gastroenterology Group, Kochi, IND; 2 Pathology, PVS Memorial Hospital, Ernakulam, IND; 3 Gastroenterology, Ernakulam Medical Center, Cochin, IND

**Keywords:** sinusoidal obstruction, veno-occlusive disease, vod, cirrhosis, portal hypertension, histopathology, ayurveda, herbal, naturopathy, cam

## Abstract

We present the rare case of a young male with sinusoidal obstruction syndrome due to Ayurvedic herbal medicine which he took for management of bilateral leg swelling associated with protein-losing enteropathy due to intestinal lymphangiectasia. The patient developed progressive sinusoidal fibrosis leading to cirrhosis on long term follow-up. In a diagnosis that took three years to conclude, we showcase serial liver biopsies that reveal the rare disease progression. Complementary and alternative medicine use among apparently healthy population is a potentially modifiable risk factor for liver diseases, in the presence of adequate public health education from concerned authorities.

## Introduction

Sinusoidal obstruction syndrome (SOS), previously known as veno-occlusive disease (VOD), is a rare and potentially fatal form of hepatic injury that occurs predominantly, after drug or toxin exposure and can present as acute, subacute or chronic form usually with jaundice, abdominal pain and swelling in the former and with evidence of portal hypertension in the latter [1]. Drugs causing SOS include busulfan, melphalan, thiotepa, cyclophosphamide, carmustine, dacarbazine, and the platinum-based carboplatin, cisplatin and oxaliplatin. The thiopurines can cause SOS rarely when given in high doses. Most important are the pyrrolizidine alkaloids which are constituents of many herbs (for example, Crotalaria species or bush tea) well-known to cause SOS. With the advent of increasing use of complementary and alternative medicine world over, herbal medicine-related incidence of liver disease is on the rise [1, 2]. We report the case of a young boy with Ayurveda herbal medicine use who developed SOS, that in due course, progressed to cirrhosis, a difficult diagnosis to make at the outset.

## Case presentation

**Figure 1:**
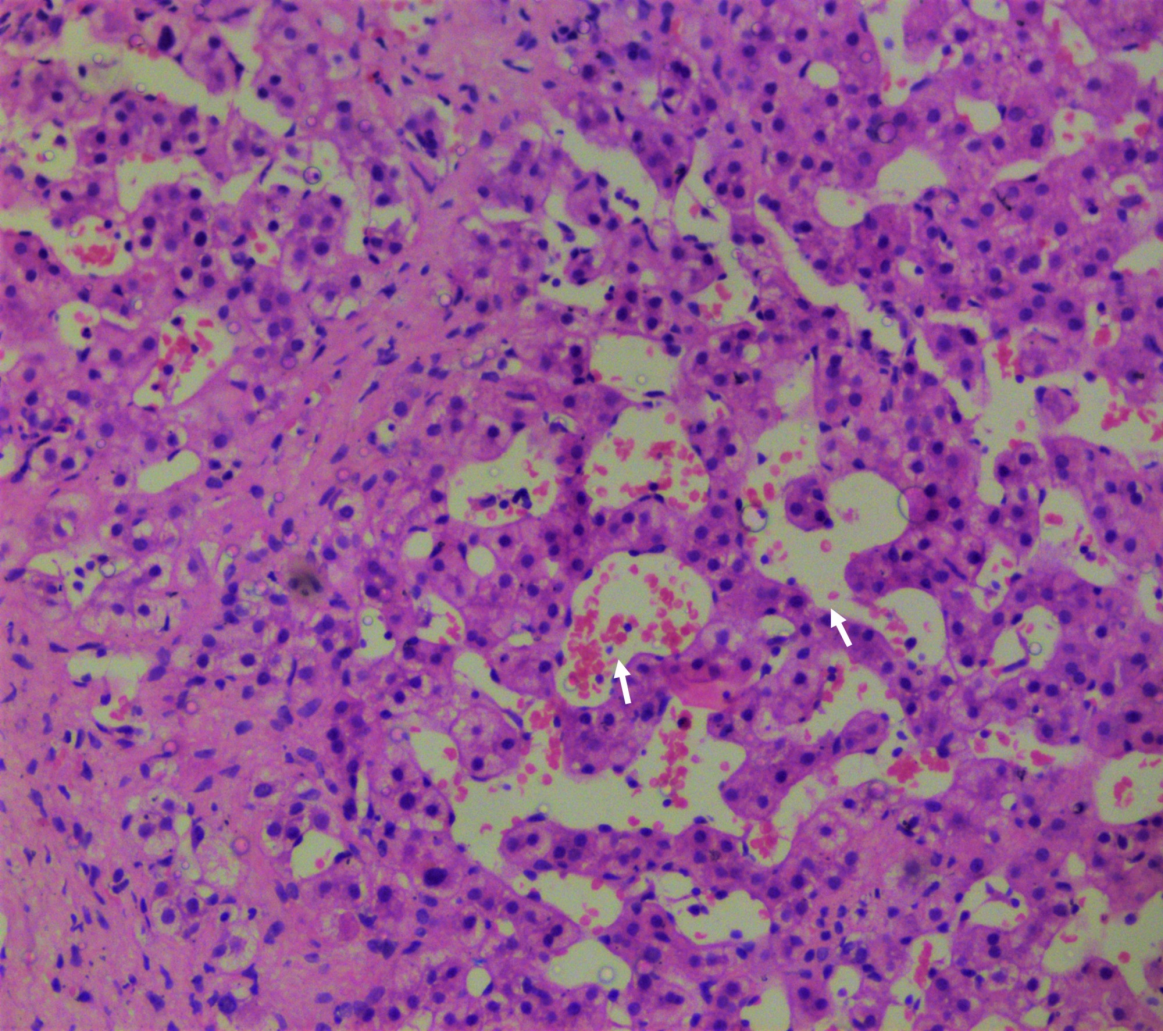
Liver biopsy findings during index presentation. Extensive sinusoidal dilatation (arrows) with few areas showing necrosis (hematoxylin and eosin stain, 20x).

**Figure 2:**
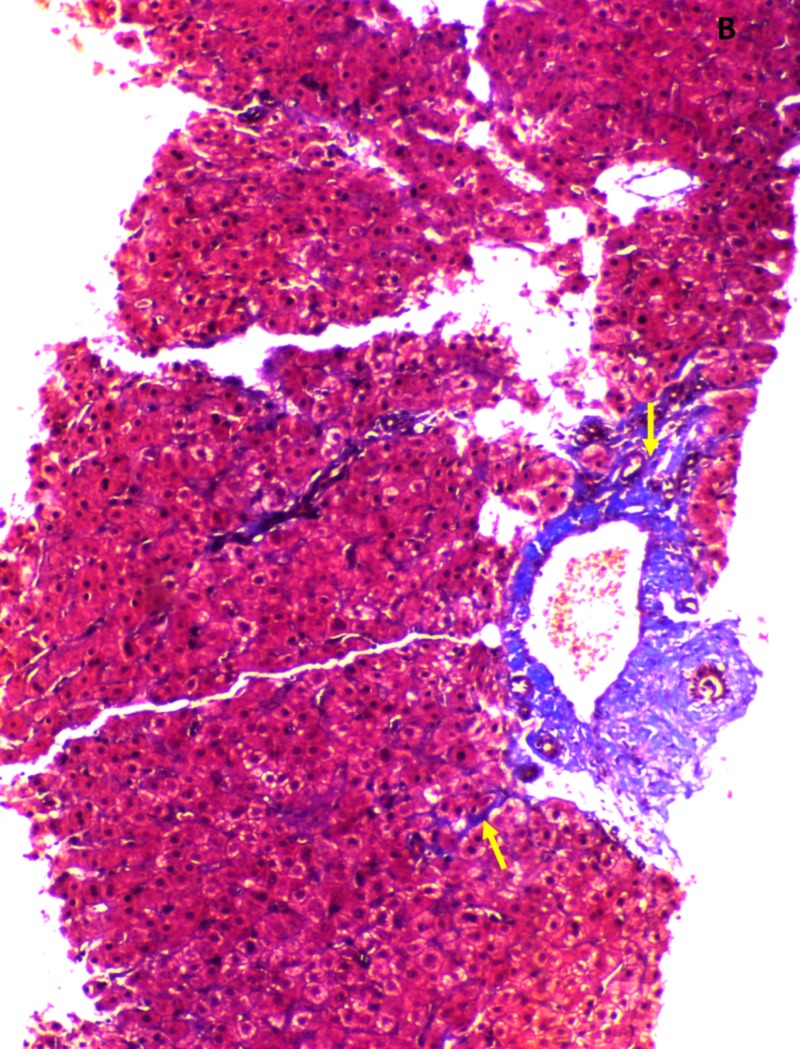
Liver biopsy findings during index presentation. Perivenular and perisinusoidal (arrows) fibrosis (Masson’s trichrome stain, 10x).

A 19-year-old boy, suffering from bilateral lower limb swelling for six months, three years ago, was found to have hypoproteinemia on blood investigation. On upper gastrointestinal endoscopic evaluation and thereafter, on push enteroscopy, he was found to have extensive duodeno-jejunal lymphangiectasia, which was confirmed on small bowel biopsy. In view of waxing and waning of lower limb symptoms, he ingested a polyherbal Ayurvedic medicine twice daily for 10 days from a traditional Ayurveda practitioner. Two weeks after consuming the complementary and alternative medicine, he developed anasarca and mild jaundice with total bilirubin 4.8 mg/dl (normal, 0.8–1.2) associated with elevation of aspartate aminotransferase 253 U/L (normal, up to 43 U/L) and alanine aminotransferase 118 U/L (normal, up to 40 U/L). The serum alkaline phosphatase was 114 U/L (normal, up to 145 U/L), serum albumin 2.6 g/dl (normal, 3.5 to 5.5 g/dl) and total protein 4.8 g/dl (normal, 6 to 8 g/dl). Contrast imaging of the abdomen revealed hepatomegaly with patchy liver enhancement and ascites without hepatic vein or inferior vena-cava obstruction. Evaluation for acute hepatotropic and non-hepatotropic viruses including Herpes virus infection and chronic viral hepatitis, Wilson’s disease and autoimmune hepatitis was non-contributory. Family history of liver disease was absent and mutational studies for hemochromatosis, alpha-1 anti-trypsin deficiency and adiponutrin were non-contributory. R ratio for identification of type of liver injury was more than five, suggestive of hepatocellular pattern. The Roussel Uclaf Causality Assessment Method (RUCAM) in drug-induced liver injury (DILI) score was eight, suggestive of probable adverse drug reaction. Liver biopsy showed extensive sinusoidal dilatation with mild perivenular, sinusoidal and perisinusoidal fibrosis (Figures 1, 2).

**Figure 3:**
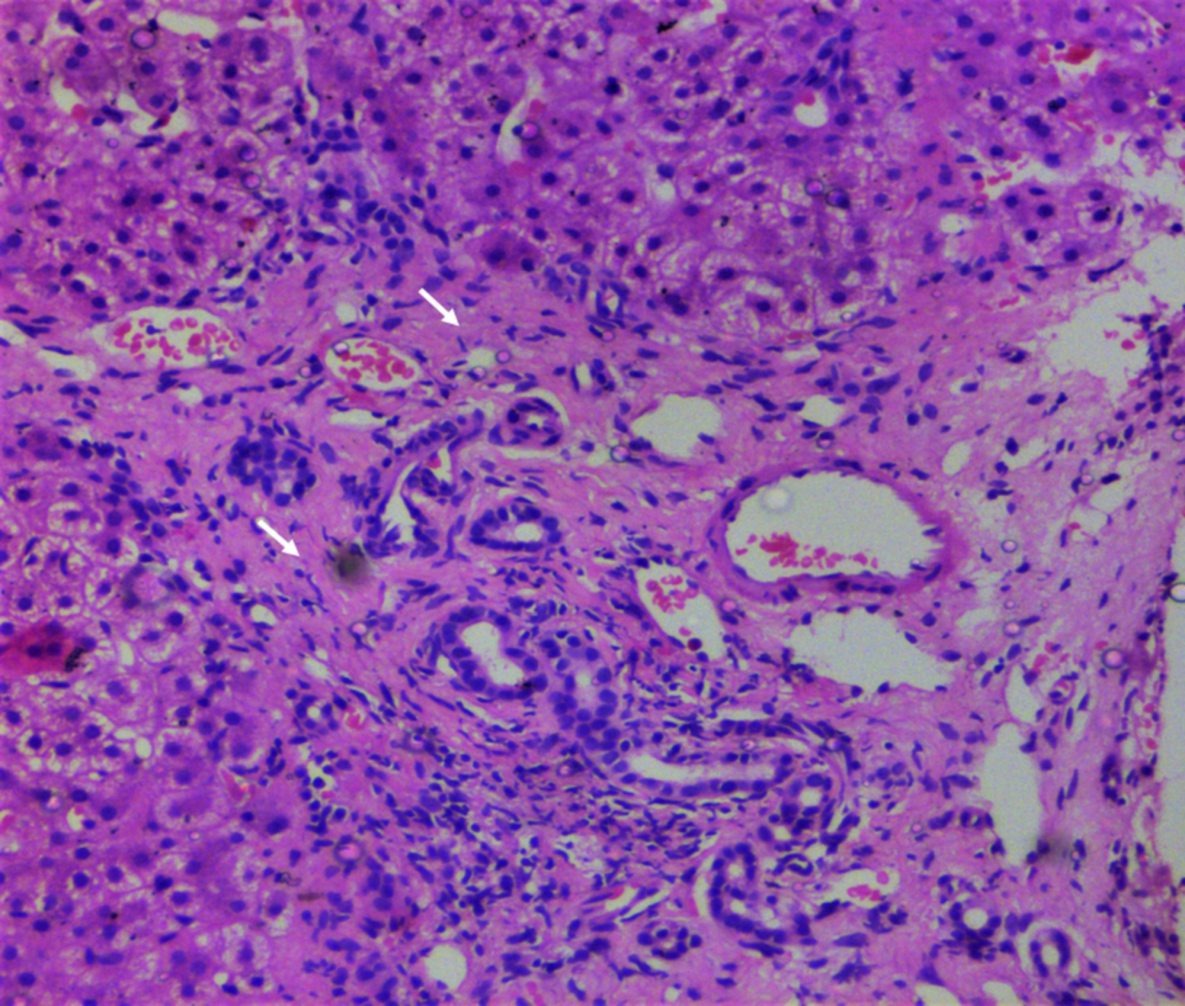
Liver biopsy findings during follow-up. Extensive sinusoidal fibrosis with minimal sinusoidal dilatation (arrows, hematoxylin and eosin stain, 20x).

**Figure 4:**
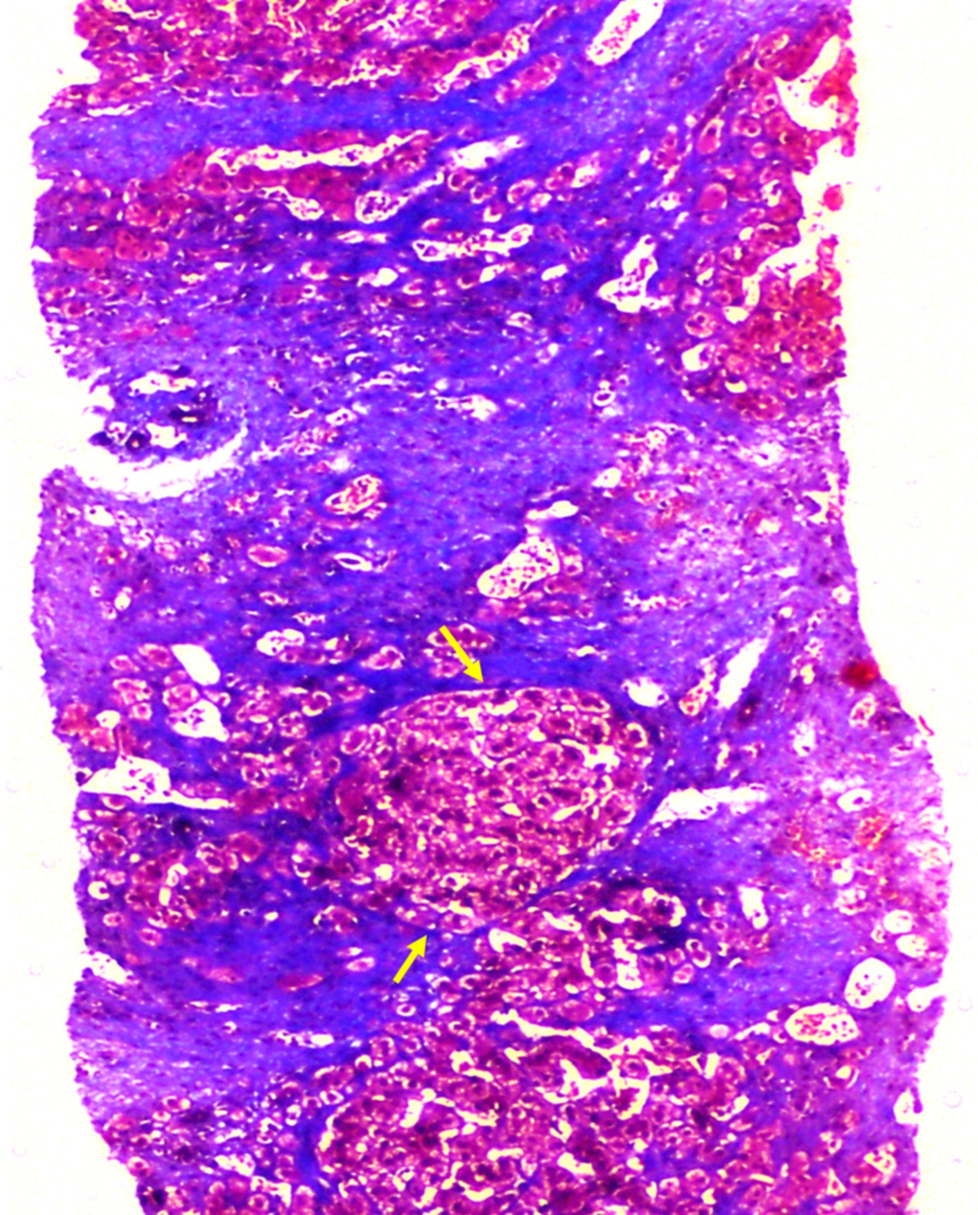
Liver biopsy findings during follow-up. Diffuse sinusoidal and perivenular fibrosis with extension into the parenchyma leading to nodule formation (arrows) suggestive of cirrhosis (Masson’s trichrome stain, 10x).

Clinical improvement was observed over 12 weeks but the patient was lost to follow up. A couple of years later, he presented to our department with ascites and worsening pedal edema. Blood investigations were significant for hypoalbuminemia and raised serum globulins without evidence of hyperbilirubinemia or coagulation failure. A repeat liver biopsy revealed dense sinusoidal and terminal hepatic perivenular fibrosis with parenchymal extension and nodule formation suggestive of chronic SOS-associated cirrhosis (Figures 3, 4).

## Discussion

Sinusoidal obstruction syndrome is a rare disease that is associated with hematopoietic stem cell and solid organ transplantation, cytotoxic chemo-radiation and immunodeficiency and most importantly, drugs and toxins. In the early stages, the disease may be focal in nature [1]. In severe acute phase, liver histopathology shows centrilobular congestion and hepatocellular necrosis. Clinically, in the acute phase, the patients may present with only lower limb edema and ascites or severe anasarca, depending on the severity of liver injury. Over a period of few weeks, fibrous scar development takes place in and around the affected terminal hepatic venule, leading to progressive venular obliteration [2]. With persistence of the lesion, dense perivenular fibrosis that later project into the hepatic parenchyma develops, rarely evolving into cirrhosis. The main differential diagnoses include other causes of venous outflow obstruction such as Budd–Chiari syndrome and right heart failure and graft versus host disease and other forms of DILI and atypical forms of viral hepatitis such as herpes simplex [1, 2]. Patients who seldom survive the acute episode, progress to chronic SOS, leading to complications of portal hypertension, but rarely cirrhosis [3]. Herbal medicines are an extremely rare cause of chronic SOS leading to cirrhosis. Plant-derived-pyrrolizidine alkaloids have been implicated in sinusoidal endothelial cell injury in earlier reports causing SOS [4]. Licinio et al. described a 17-year-old girl with intestinal lymphangiectasia and progressive fibrosis of the liver not related to any identifiable etiology. The liver biopsy in their report revealed pericellular and periportal fibrosis interpreted as “congenital liver fibrosis”. In our patient, the liver biopsy was typical and progression classical of SOS with RUCAM suggestive of probable DILI [5]. Pseudo-scientific-health-seeking behaviour aimed at ‘safer’ alternatives, and associated public health promotion of pseudo-science, instead of discouragement, from Government agencies in countries entrenched in ancient medical practices are great concerns [6]. Our patient is currently on management of portal hypertension and on follow-up without complications of liver failure.

## Conclusions

A rigorous drug history, especially of alternative medicine use, is mandatory when caring for a liver-disease patient. Public health education and partnerships that integrate complementary and alternative medical practitioners into surveillance programs are unmet needs in countries entrenched in traditional medical practices.

## References

[1] Mohty M, Malard F, Abecassis M (2015). Sinusoidal obstruction syndrome/veno-occlusive disease: current situation and perspectives-a position statement from the European Society for Blood and Marrow Transplantation (EBMT). Bone Marrow Transplant.

[2] Fan CQ, Crawford JM (2014). Sinusoidal obstruction syndrome (hepatic veno-occlusive disease). J Clin Exp Hepatol.

[3] Zhou H, Wang YX, Lou HY, Xu XJ, Zhang MM (2014). Hepatic sinusoidal obstruction syndrome caused by herbal medicine: CT and MRI features. Korean J Radiol.

[4] Valla DC, Cazals-Hatem D (2016). Sinusoidal obstruction syndrome. Clin Res Hepatol Gastroenterol.

[5] Licinio R, Principi M, Ierardi E, Di Leo A (2014). Liver fibrosis in primary intestinal lymphangiectasia: an undervalued topic. World J Hepatol.

[6] Shrivastava SR, Shrivastava PS, Ramasamy J (2015). Mainstreaming of Ayurveda, Yoga, Naturopathy, Unani, Siddha, and Homeopathy with the health care delivery system in India. J Tradit Complement Med.

